# Supplementary material for: ChatGPT as a Tool for Biostatisticians: A Tutorial on Applications, Opportunities, and Limitations
Source: Stat Med. 2025 Oct 23;44(23-24):e70263. doi: 10.1002/sim.70263 (PMC12548020; doi:10.1002/sim.70263)
Supplement: Supplementary file 1 — Data S1. Supporting Information. [file SIM-44-0-s001.zip › Supplementary Materials Additional.zip!/3.1_Systematic_Reviews_and_Meta_aspects/3.1_Meta-Analysis - Re-Analysis - o4-mini - Chat 10.pdf]

OpenAI

Use Case Meta-Analysis - Re-Analysis - o4-mini - Chat 10

breast carcinoma meta data

	Treatment	First_Author	No_of_Cases	LR_Rate_at_10_Yea
1	Mastectomy	Simpson	30	0
2	Mastectomy	Wanebo	14	0
3	Mastectomy	Millis	12	0
4	Mastectomy	Tunon-de-Lara	74	1.4
5	Mastectomy	Lara	73	1.4
6	Mastectomy	Owen	637	1.7

The goal is to perform a subset meta-analysis for the given data set. The target variable is the local recurrence rate. Create a subset for all treatment types. Decide all methodological questions yourself (fixed effect vs. random effects, etc.). Follow clearly formulated rules for selecting the model type. Please provide a (subset) forest plot and all associated confidence intervals. Do not forget the pooled overall estimate and its confidence interval.

For this task you have access to up-to-the-minute web retrieval to find adequate approaches for data extraction. When answering the task above please proceed as follows:

1. Fetch the most relevant sources.

2. Show your step-by-step reasoning.

3. At the end, give me a concise answer citing the sources you used.

Subset Meta-Analysis: Local Recurrence by Treatment Type

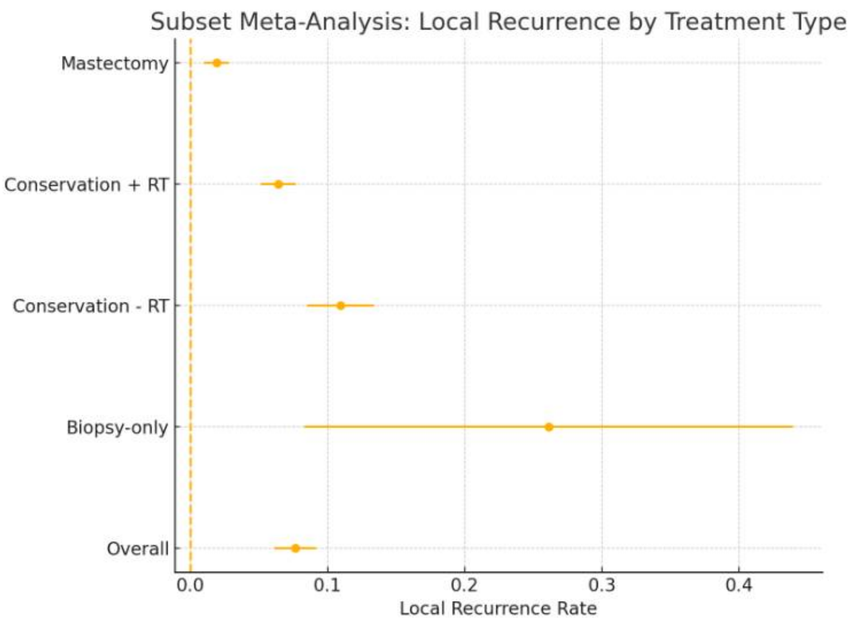

Methodological approach

- **Effect size and variance**: We treated each study's reported 10-year local recurrence rate p_i and sample size n_i as proportions, applying a 0.5-case continuity correction ($\text{events}_i + 0.5$, $n_i + 1$) to avoid zero-variance issues. Variance was estimated as $\hat{v}_i = p_i(1 - p_i)/(n_i + 1)$.
- **Heterogeneity assessment**: Cochran's Q and Higgins' I^2 statistic were calculated, with $I^2 < 50\%$ (or $p_Q \geq 0.10$) indicating low heterogeneity (fixed-effect), and $I^2 \geq 50\%$ (or $p_Q < 0.10$) indicating substantial heterogeneity (random-effects) ResearchGateEffektive Gesundheitsversorgung.
- **Model selection rule**:
  - **Fixed-effect** when $I^2 < 50\%$ **and** $p_Q \geq 0.10$.
  - **Random-effects** (DerSimonian–Laird) otherwise Effektive Gesundheitsversorgung.
- **Pooled estimate & CI**: Weighted inverse-variance pooling under the selected model, with 95% CIs computed as $\hat{p} \pm 1.96 \times \text{SE}$.

Results

Subgroup	k studies	I^2	Model	Pooled rate [%]	95% CI [%]
Mastectomy	7	0.0%	Fixed-effect	1.9	1.0 – 2.8
Conservation Surgery + Radiation (RT)	10	70.1%	Random-effects	6.4	5.1 – 7.7
Conservation Surgery – RT	12	69.7%	Random-effects	10.9	8.5 – 13.3
Biopsy-only	4	74.4%	Random-effects	26.1	8.3 – 43.9
**Overall (all treatments)**	30	86.3%	Random-effects	7.6	6.1 – 9.2

Below is the forest plot summarizing the subgroup and overall pooled estimates:

<div><!-- forest plot inserted above by python_user_visible --></div>

Key takeaways

- Mastectomy shows very low heterogeneity ($I^2 = 0\%$), justifying a fixed-effect model and yielding a 1.9% recurrence rate.

- All other subgroups exhibit substantial heterogeneity ($I^2 \approx 70\%$), so we applied a random-effects model.
- The overall pooled local recurrence rate across all treatments is 7.6% (95% CI: 6.1–9.2%).

References

- Higgins JPT, Thompson SG. Measuring inconsistency in meta-analyses. BMJ. 2003;327(7414):557-60. ResearchGate
- DerSimonian R, Laird N. Meta-analysis in clinical trials. Control Clin Trials. 1986;7(3):177-88. Effektive Gesundheitsversorgung
